# Attenuation of photosynthesis in nanosilver-treated *Arabidopsis thaliana* is inherently linked to the particulate nature of silver

**DOI:** 10.1016/j.heliyon.2024.e27583

**Published:** 2024-03-07

**Authors:** Vincent Mays, Natalie Smith, Cody Pham, Margaret White, Qihua Wu, Jacob Berry, Alexander Linan, D. Alexander Wait, Laszlo Kovacs

**Affiliations:** a Department of Biology, Missouri State University, Springfield, MO, USA; b Jordan Valley Innovation Center, Missouri State University, Springfield, MO, USA; c Missouri Botanical Garden, St. Louis, Missouri, 63110, USA

**Keywords:** Nanosilver, Silver ions, Arabidopsis, Chlorophyll, Photosynthesis, Phytotoxicity, RNA-Seq

## Abstract

Silver nanoparticles (AgNPs) are known to affect the physiology and morphology of plants in various ways, but the exact mechanism by which they interact with plant cells remains to be elucidated. An unresolved question of silver nanotoxicology is whether the interaction is triggered by the physical features of the particles, or by silver ions leached from their surface. In this study, we germinated and grew *Arabidopsis thaliana* seedlings in synthetic medium supplemented with sub-morbid concentrations (4 μg/mL) of AgNPs and silver nitrate (AgNO_3_). This treatment led to *in planta* accumulation of 106 μg/g and 97 μg/g of silver in the AgNO_3_- and AgNP-exposed seedlings, respectively. Despite the statistically indistinguishable silver accumulation, RNA sequencing data demonstrated distinct changes in the transcriptome of the AgNP-exposed, but not in the AgNO_3_-exposed plants. AgNP exposure induced changes in the expression of genes involved in immune response, cell wall organization, photosynthesis and cellular defense against reactive oxygen species. AgNO_3_ exposure, on the other hand, caused the differential expression of only two genes, neither of which belonged to any AgNP-enriched gene ontology categories. Moreover, AgNP exposure led to a 39% reduction (p < 0.001) in total chlorophyll concentration relative to untreated plants which was associated with a 56.9% and 56.2% drop (p < 0.05) in carbon assimilation rate at ambient and saturating light, respectively. Stomatal conductance was not significantly affected by AgNP exposure, and limitations to carbon assimilation, as determined through analysis of light and carbon dioxide (A/Ci) curves, were attributed to rates of electron transport, maximum carboxylation rates and triose phosphate use. AgNO_3_-exposure, on the other hand, did not lead to significant reduction either in chlorophyll concentration or in carbon assimilation rate. Given these data, we propose that the impact of AgNPs cannot be simply attributed to the presence of the metal in plants, but is innate to the particulate nature of nanosilver.

## 1 Introduction

The manufacturing and application of engineered nanomaterials (ENMs), particles that are less than 100 nm in at least one dimension, are gaining momentum at a rapid pace [1]. Silver nanoparticles (AgNPs) are among the most widely used metal-based ENMs. To utilize their unique physicochemical, electronic, optical, and antimicrobial properties, AgNPs are incorporated into electronic and biomedical devices, paints, textiles, food packaging, hygiene products, and cosmetics [2]. The use of AgNPs as biostimulants, nanopesticides, and nanofertilizers on crops is considered a viable option to improve agricultural production (reviewed by Ref. [3]. As the volume of production and breadth of applications of AgNPs expand, so does their environmental release in the form of agricultural run-off, wastewater effluent and consumer solid waste [4,5]. To properly assess the risk associated with nanosilver pollution, it is essential to have an understanding of how AgNPs interact with living organisms.

AgNPs have been shown to be both beneficial and harmful to plants, in a particle size-, particle concentration- and plant species-dependent manner, but the exact mechanisms of their interaction with plant cell components remain to be elucidated [6]. AgNPs are thought to primarily enter plants through the roots, in a size-dependent manner. It has been shown that AgNPs of <40 nm-size are able to pass directly through the cell wall and be transported through the shoot system [7,8]. The harmful effects of *in planta* AgNPs has been demonstrated at the morphological level by the reduction in root and shoot length and the decrease in biomass [9,10]. At the physiological level, AgNP exposure has been shown to reduce chlorophyll and carotenoid contents and decrease photosynthesis [[9], [10], [11]]. At the cellular level, AgNPs damage cytoplasmic membranes and the cell wall, cause plasmolysis and vacuolization, and induce malformations in chloroplasts and mitochondria [[12], [13], [14]]. Results from numerous studies are in agreement that AgNPs trigger an elevated level of reactive oxygen species (ROS) (reviewed in Refs. [6,15,16]). However, Ag^+^ ions, which are known to leach from AgNPs [17], have also been shown to induce ROS production in a similar way [12]. This raises the possibility that silver nanotoxicity may be due to Ag ^+^ dissociated *in planta* from the surface of AgNPs, rather than its particulate nature. AgNP dissolution is well studied and depend upon several environmental factors including pH, ionic strength, redox conditions, and organic matter [[18], [19], [20], [21], [22], [23], [24]]**.**

In this study, we sought to test the hypothesis that nanosilver phytotoxicity is linked to the particulate nature of silver. To assess this, we exposed *Arabidopsis thaliana* seedlings to equal sub-morbid concentrations (4ug/mL) of either AgNPs or AgNO_3_. This mild treatment avoids triggering excessive levels of ROS, which themselves are stress factors and could mask the impact of the primary stressor. We also minimized dissociation of Ag^+^ ions from AgNPs by buffering the pH of the medium at 7.0. If Ag^+^ alone is responsible for phytotoxicity, then the accumulation of equal quantities of Ag in plants would be expected to induce comparable changes in gene expression and physiological functions. Though similar experiments have been reported previously [3,10,11,13,[25], [26], [27]], our approach differs in that we ensured similar *in planta* Ag concentrations in both nanosilver and Ag ^+^ treatments, and that both our transcriptomic and physiological data were generated from independently repeated experiments as opposed to biological replicates.

## 2 Materials and methods

### 2.1 Nanoparticles, culture media, and experimental design

The AgNPs used in this study were 20 nm-diameter quasi-spherical neutral silver core particles stabilized by a citrate shell. They were purchased as a colloidal preparation in 2 mM sodium citrate (PELCO® NanoXactTM particles) from Ted Pella, Inc. (Redding, California, USA). The size of AgNPs was validated with dynamic light scattering (DLS) using a Nano-flex II instrument (Colloid Metrix, Inning, Germany). Treated plants were grown axenically in culture medium supplemented with AgNPs or AgNO_3_. Control plants were grown in non-modified medium. The culture medium was composed of half-strength Murashige and Skoog nutrients with Gamborg's vitamins supplemented with 2.5% MOPS buffer and 0.8% agar. The final pH was adjusted to 7.0 using 100 mM KOH. The medium was sterilized in an autoclave at 121 °C for 20 min. Once cooled to 55 °C, it was supplemented with sterilized water (control), AgNP suspension or AgNO_3_ solution for a final concentration of 4 μg/mL. After adding the supplement, the medium was sonicated for 2 min to prevent particle aggregation. Carbenicillin (50 μg/mL) and amphotericin B (2.5 μg/mL) were added to ensure axenic growth conditions. Plants were grown in blocked environments (Petri dishes) in three technical replicates within each biological repeat.

### 2.2 Plants and culture conditions

The experiments were performed on *A. thaliana* Col-0 ecotype. Seeds were sterilized for 3 h in chlorine gas generated by combining 3 mL of concentrated HCl with 100 mL of 6% NaOCl (Clorox bleach). The sterile seeds were then pipetted in a 10 x10 grid on the surface of the agar-solidified plant culture media in Petri plates under axenic conditions. The plates were then wrapped with Parafilm, and the seeds stratified at 4 °C in the dark for 3 days. Following stratification, the Parafilm seal was removed, the plates were placed in unsealed sandwich-sized plastic bags and transferred to a Conviron Adaptis A1000-AR Growth Chamber (Winnipeg, Manitoba, Canada) for 14 days (21 days for gas exchange measurements). Plants were grown at 25 °C, in a 10-h light/14-h dark diurnal cycle. During the entire growth period, the plates were randomly rearranged daily in the growth chamber to mitigate positional effect.

### 2.3 Carbon assimilation measurements

Gas exchange was measured in nine replicates per biological repeat using a LI-6400 XT portable photosynthetic system with a 6 cm^2^ leaf chamber (LI-COR Biosciences at Lincoln, Nebraska, USA). The infrared gas analyzer was factory-calibrated prior to the initiation of the experiments. During measurements, the flow rate was set to 300 μmol/s and fan speed to slow. Block temperature was set to 25 °C and leaf temperatures varied between 23 and 25 °C. Relative humidity was controlled to be between 70% and 75%. A section of agar-solidified medium with complete 21- day-old seedlings was placed on filter paper. Leaf area was measured after gas exchange analysis by placing samples on a grid and determining leaf area to 0.5 cm^2^. Leaf area varied between 4 and 6 cm^2^ for all measurements. Samples were randomly chosen and measured for 30–40 min at 3–6 h after the start of the diurnal light period to allow stable photosynthetic rates, stomatal conductance, and transpiration rates. The following measurements were taken: ambient photosynthetic rate (A_amb_), photosaturated photosynthetic rate (A_max_), photosynthetic responses to photosynthetically active radiation (PAR), and photosynthetic responses to carbon dioxide (A/Ci). A_amb_ is the rate of carbon assimilation at growth light levels in the growth chamber, which was 150 μmol/m^2^/s at growth CO_2_ levels (400 μmol CO_2_/m^2^/s). Measuring at growth, PAR indicates the efficiency at which the seedling assimilates CO_2_ in the chamber during its growth. A_max_ is the rate of carbon assimilation at saturating light (PAR of 500 μmol/m^2^/s). For determining photosynthetic responses to PAR, data were recorded at five light levels (0, 50, 150, 300, and 500 μmol/m^2^/s) and with CO_2_ maintained at 400 μmol CO_2_/m^2^/s. For determining photosynthetic responses to CO_2_ level, data were recorded at five light levels (0, 50, 200, 400, and 700 μmol CO_2_/m^2^/s), and light intensity was maintained at 500 μmol/m^2^/s for A/Ci curve measurements. A curve-fitting program developed by Sharkey [28] was used to estimate the maximum rate of electron transport at saturating light (J_max_), the initial slope of J (Φ), and the convexity factor (Θ). Light response curve analysis was done on the basis of the following data: leaf temperature (T_leaf_), atmospheric pressure (P_atm_), day respiration (R_d_), ambient O_2_, mesophyll conductance (g_m_), photosynthesis rate (A), intracellular concentration (Ci), and light intensity. R_d_ was assigned as the datum point measured at the lowest light intensity (PAR = 0 μmol/m^2^/s). P_atm_ = 97.7 kPa at 304 m elevation, O_2_ = 21 kPa, and g_m_ = 2 μmol/m2/s Pa-1 were kept constant for all treatments.

### 2.4 Chlorophyll extraction and concentration measurement

Following the 14-day growth period, leaves were harvested and then stored at −80 °C until processing. Tissue was homogenized in liquid nitrogen, suspended in 5 mL of 80% acetone solution in light-protected 15-mL Falcon tubes and incubated in the dark for 30 min. Following centrifugation (15 min, 3000 rpm at 4 °C), the supernatant was transferred into a fresh, light-protected Falcon tube. One mL of the supernatant was removed from each sample and a BioMate 3 spectrometer (Thermo Fisher Scientific, Madison, Wisconsin, USA) was used to measure absorbance values at 663 nm and 645 nm. The equations of Arnon [29] were used to calculate the concentration of total chlorophyll and of chlorophyll A and B.

### 2.5 Inductively coupled plasma optical emission spectroscopy (ICP-OES) analysis

The concentration of Ag and 9 other elements in the plants was quantified using an inductively coupled plasma-atomic emission spectrometry (ICP-OES) instrument (Thermo Scientific iCAP7400 Duo). Plant shoot system and whole plants were rinsed with DI water and dried at 50 °C for 4 days. Dried samples of 0.01–0.2 g were transferred into open glass vials and wet-digested by 3 mL of 70% nitric acid at 130 °C for 1 h, followed by 1 mL of hydrogen peroxide (30%) at 130 °C for a subsequent hour. Digested samples were diluted to a volume of 5 mL with DI water and filtered by 0.45 μm PTFE filter prior to test. ICP-OES analyses were conducted according to Environmental Protection Agency method 200.7 [30] with minor modifications. Reagent blank and standard solutions with known amounts of standard mixtures were used for quality control (QC) purposes. The accuracy of the test method was monitored by analyzing QC standards for every 10 samples. The method was validated in respect of method detection limit (MDL), linearity range and repeatability. Spike recovery was determined by adding known amounts (50 μg/g to 1000 μg/g) of analytes into sample matrices and the response was measured by ICP-OES and compared to the identical spike concentrations in the standard solution. Average percent recoveries are reported in Table 1.

**Table 1:** Concentration of silver and nine other elements in Arabidopsis seedlings grown in 4 μg/mL AgNP- or 4 μg/mL AgNO3-supplemented medium across three biological replicates. Spike recovery was determined by adding a known amount (50 μg/g to 1000 μg/g) of analytes into sample matrices. Each response was measured by ICP-OES and compared to the identical spike concentrations in the standard solution. Each value represents the mean ± SD of three technical replicates.

Elements	Plants Grown on AgNP Medium			Plants Grown on AgNO3 Medium			Spike Recovery (%)
	(μg/g dry plant mass)			(μg/g dry plant mass)			50–1000 μg/L
	Rep 1	Rep 2	Rep 3	Rep 1	Rep 2	Rep 3	
Ag	87.25 ± 22.21	106.06 ± 24.23	97.67 ± 4.86	102.05 ± 18.86	115.12 ± 22.61	100.60 ± 11.61	97.27 ± 3.55
Zn	126.52 ± 11.79	132.65 ± 23.69	117.59 ± 11.89	144.32 ± 10.51	156.07 ± 4.10	148.29 ± 19.32	96.20 ± 4.43
Fe	91.30 ± 20.01	93.10 ± 4.04	83.44 ± 6.68	109.70 ± 5.57	100.71 ± 5.43	95.96 ± 7.91	92.58 ± 1.26
Al	186.39 ± 38.76	185.75 ± 62.11	136.55 ± 29.26	231.66 ± 144.98	260.54 ± 91.35	199.28 ± 90.53	98.14 ± 2.09
Cu	9.93 ± 0.94	11.34 ± 4.18	16.95 ± 13.79	19.96 ± 9.12	27.52 ± 16.04	30.83 ± 18.48	91.56 ± 2.30
K	40971.46 ± 3101.62	42264.10 ± 708.44	39360.88 ± 6544.85	47675.10 ± 1286.59	42537.20 ± 1410.77	42298.68 ± 1812.64	98.17 ± 4.02
Mg	2323.37 ± 120.13	2454.74 ± 185.98	2182.54 ± 415.63	2683.45 ± 218.41	2711.85 ± 307.19	2410.17 ± 146.17	109.65 ± 10.32
Mn	247.69 ± 72.26	259.56 ± 48.71	192.91 ± 4.07	218.90 ± 53.23	217.87 ± 17.05	251.48 ± 32.53	109.35 ± 8.67
Na	15509.43 ± 1152.67	16845.23 ± 1417.84	15846.31 ± 1337.39	14259.73 ± 1787.16	14388.67 ± 1112.69	12007.63 ± 1671.91	121.53 ± 11.62
Ca	3939.30 ± 325.26	4225.20 ± 238.10	3758.17 ± 666.93	4767.56 ± 318.15	4804.17 ± 510.45	4269.28 ± 212.54	115.52 ± 8.76

### 2.6 RNA extraction, RNA-seq library construction and sequencing

RNA extraction and RNA-seq analysis were done at two separate times, first for control vs. AgNPs and later for control vs. AgNO_3_, due to budgetary constraints. Total RNA was extracted from 14-day-old plants using the Trizol reagent (Invitrogen, Carslbad, California, USA). In all experiments, RNA extraction was performed 3 h after the start of the light period to mitigate the effect of the diurnal cycle on gene expression. RNA samples were further purified using the RNeasy Extraction Kit by Qiagen (Hilden, Germany) following the manufacturer's guidelines. mRNA purification and RNA-seq library construction were performed using the TruSeq Stranded mRNA Sample Preparation Kit by Illumina Corporation (San Diego, California, USA) following the low sample-size protocol. In brief, poly(A) mRNA was selected from 1 μg of total RNA using poly(T) beads. The selected mRNA was fragmented, purified, and reverse-transcribed into cDNA. The cDNA underwent end-repair, adapter ligation, and strand selection to generate stranded sequencing libraries. The libraries were purified, amplified, and sequenced (100-bp read-length) on two flow cells of an Illumina HiSeq 2500 Sequencing System at the Genome Sequencing Facility of the University of Kansas Medical Center.

### 2.7 RNA-seq analysis

RNA-seq analysis for differential gene expression was performed using the bioinformatics software CLC Genomics Workbench v7.0.4, v9.5.1. and v20.0.4 (Qiagen Bioinformatics, Redwood City, California, USA). Samples were imported as paired reads, then filtered based on length (between 15 and 1000 nt) and quality (limit 0.05) using default parameters. Fifteen nucleotides were deleted from the 5’ end of all reads to remove any remaining adapter sequences. Reads were mapped to the *A. thaliana* TAIR10 reference genome sequence, downloaded from the ENSEMBL database using default parameters in the forward direction. Expression data from mapped reads were normalized as the number of reads per kilobase per million reads mapped (RPKM). Differentially expressed genes (DEGs) were identified through pairwise comparison of control and AgNP treatment libraries. DEGs were defined as transcripts that were at least 2-fold up- or down-regulated at a p-value of less than 0.05.

### 2.8 Real-time quantitative PCR (RT-qPCR) analysis

To independently confirm the results of our RNA-seq analyses, we selected eight DEGs identified by RNA-seq and performed RT-qPCR on RNA samples from an independently repeated experiment as well as the same RNA samples that were subject to RNA-seq. Genes AT3G18780 or AT4G02080 were used as reference to normalize expression. cDNA synthesis was performed using the SuperScript® II Reverse Transcriptase kit, the SuperScript™ IV Reverse Transcriptase Kit or the First-Strand cDNA Synthesis Kit from Invitrogen using 1 μg of RNA and random primers at 100 ng/μL concentration. Primer pairs were designed based on sequence data obtained from The Arabidopsis Information Resource (TAIR) database [31]. qPCR experiments were performed using the GoTaq qPCR Master Mix (Promega Corp., Madison, Wisconsin) or Power SYBR Green PCR Master Mix (Applied Biosystems, Dublin, Ireland). Primer efficiency for each primer pair was determined on a series of 5-fold cDNA dilutions in quadruplicates.

### 2.9 Statistical methods

Experiments were performed in three biological repeats, which were true repeats in that they were carried out sequentially and independently on newly prepared media and freshly sterilized batches of seed. Each biological repeat included three replicates in which 100 seedlings were grown in blocked environments (Petri dishes). For RNA-seq, chlorophyll concentration and ICP-OES measurements, experiments were performed on each replicate of each repeat individually, except that in RNA-seq individually extracted and quantitated technical replicates were pooled before library construction. In chlorophyll concentration measurements, two spectrophotometric readings were taken and the mean value was used for each replicate. Gas exchange and qPCR experiments were also performed in three biological repeats, using nine and two technical replicates per repeat in gas exchange and qPCR measurements, respectively.

In ICP-OES and gas exchange measurements, significant differences among treatment groups were tested using one-way ANOVA analysis with post-hoc Tukey honest significant difference (HSD) of pairwise comparisons. In chlorophyll concentration measurements, the mean total chlorophyll concentration of three technical replicates was calculated for AgNP, AgNO3 and control. The percent of control value was then determined for each treatment type and a pairwise comparison was performed by *t*-test. In both ANOVA and *t*-test, a p-value<0.05 was considered significant. Statistical analyses were performed using the GraphPad Prism 10.10 software (Dotmatics, Boston, Massachusetts, USA).

In RNA-seq analysis, differentially expressed genes (DEGs) were identified through pairwise comparison of control and AgNP treatment libraries using the Exact Test algorithm [32] implemented by the software Empirical Analysis of Differential Gene Expression (EDGE). To reduce the probability of type-I error, false discovery rate (FDR)- and Bonferroni-correction of DEGs were performed using EDGE. DEGs were defined as transcripts that were at least 2-fold up- or down-regulated at a p-value of less than 0.05. In qPCR analysis, the average Ct value for each gene was used for differential expression analysis using the Pfaffl equation [33].

## 3 Results

### 3.1 Size validation of AgNPs

**Fig. 1:**
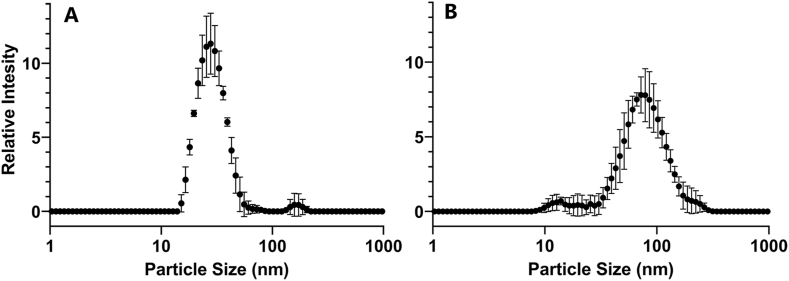
Dynamic light scattering profile of 20-nm citrate-coated silver nanoparticles dispersed at 4 μg/mL concentration in **(A)** distilled water and **(B)** MOPS-buffered half-strength MS plant growth medium.

To validate the 20-nm size of the AgNPs used in this experiment, we diluted the stock solution to 4.0 μg/mL in either water or in agar-free experimental plant medium and measured the hydrodynamic diameter of particles using DLS. In water, we were able to detect particles of 30.9 ± 11.1 nm diameter, which corresponded well to the expected hydrodynamic diameter of the AgNPs (Fig. 1A). In the liquid form of the AgNP-supplemented medium we were only able to detect trace amounts of particles in this size range and observed that the majority of the particles were in the 82.6 ± 43.2 nm diameter range (Fig. 1B). This data demonstrated that in liquid medium, and possibly in agar-solidified medium also, AgNPs aggregated into larger particles. Such aggregation of AgNPs in plant culture medium has been reported previously [34].

### 3.2 Uptake of Ag in plants grown on AgNP and AgNO_3_ media

**Fig. 2:**
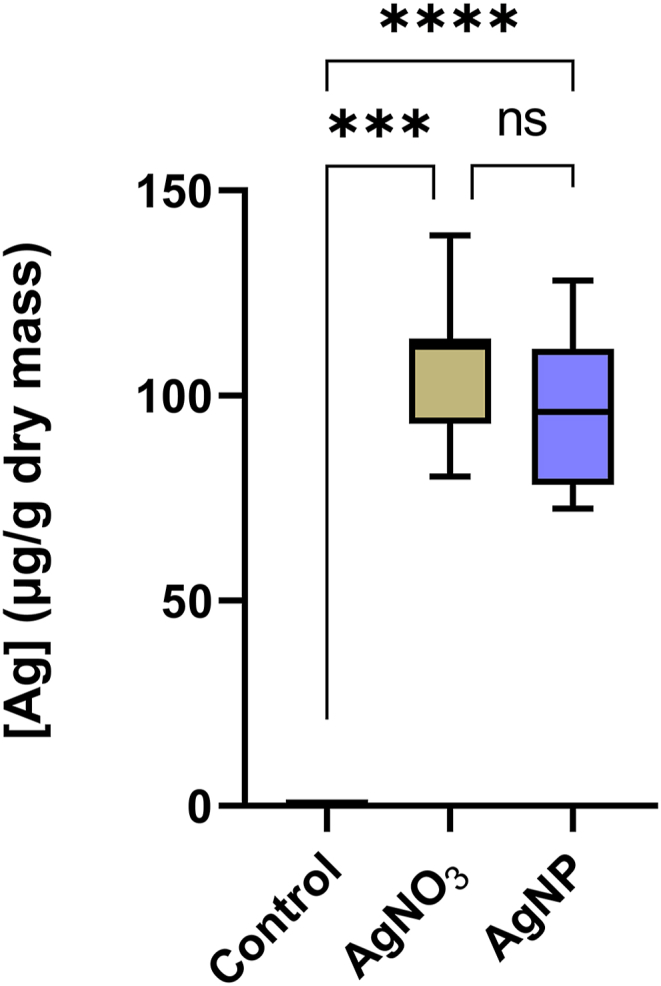
Concentration of silver (μg/g dry plant mass) in *A. thaliana* seedlings grown on media containing 4 μg/mL AgNP or 4 μg/mL AgNO3. Three technical replicates per treatment were tested with ICP-OES, with standards of known concentration run as quality control every 10 samples. ns, non-significant, *** and **** significant at *p* < 10^−3^ and p < 10^−4^, respectively.

With the aggregation of AgNPs, it was imperative to test if roots take up Ag when Arabidopsis is grown in the experimental medium. We measured Ag concentration in plants grown for 14 days on AgNP- and the AgNO_3_-containing media using ICP-OES analysis. We found that the Ag content of AgNP- and the AgNO_3_-exposed plants was 97.0 ± 19 μg/g and 106.9 ± 17 μg/g, respectively (on a plant dry mass basis), which was statistically not different (p = 0.86). We were unable to detect Ag in plants grown on control medium (Fig. 2 and Table 1). We also measured the elemental content of macro- and micronutrients K, Ca, Mg, Zn, Fe, Mn and Cu, as well as Na and Al in the AgNP- and AgNO_3_-exposed plants and found no difference in their concentrations between the two treatment groups (Table 1).

### 3.3 RNA-seq analysis

**Fig. 3:**
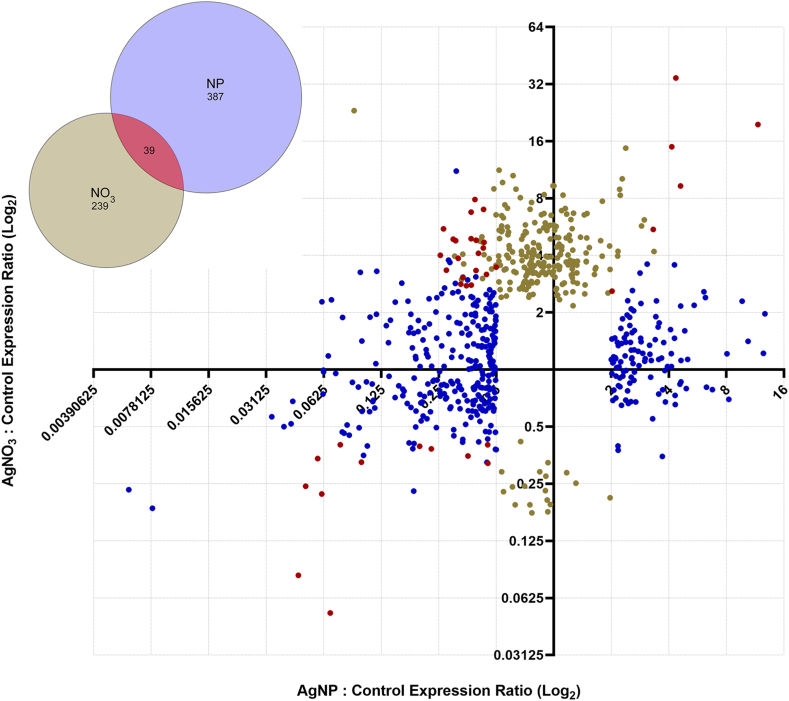
Fold-change in transcript abundance of genes differentially expressed in response to 4 μg/mL AgNP or 4 μg/mL AgNO_3_. Transcripts from three technical replicates per treatment were pooled together for library construction. Differentially expressed transcripts were those that had ≥2 or ≤ -2-fold transcript abundance of control at p ≤ 0.05. Symbols represent AgNP- and AgNO_3_-regulated DEG transcripts. The position of each transcript symbol represents its AgNP-induced fold change plotted against its AgNO_3-_induced fold-change. Transcripts that were not expressed differentially are not shown. Blue, beige, and red symbols indicate transcripts that responded to AgNPs only, AgNO_3_ only, and both AgNP and AgNO_3_, respectively. Inset: Venn diagram of the overlap between the AgNP- and the AgNO_3_-responsive gene set.

To gain insight into the impact of internalized silver on the transcriptome, we conducted a comparative RNA-seq analysis in AgNP- and AgNO_3_-exposed Arabidopsis plants. A summary of the sequencing and read-mapping data are shown in Table 2. We considered a gene significantly up- or down-regulated if its transcript abundance changed at least two-fold up or down in response to AgNP or AgNO_3_ treatment at p ≤ 0.05. We refer to such genes as differentially expressed genes (DEGs). AgNP and AgNO_3_ uptake caused the significant up- or down-regulation of 426 and 278 genes, respectively, with 39 genes shared between the two DEG sets (Fig. 3 and Supplemental Files 1 and 2). Upon false discovery rate (FDR) correction, however, the number of DEGs was reduced to 65 and 2 in the AgNP- and AgNO_3_-exposed plants, respectively (Supplemental Files 1 and 2). RNA-seq results were validated in eight AgNP-responsive and two of the AgNO_3_-responsive DEGs. qPCR analysis confirmed that the transcript abundance of all ten genes changed in the same direction as in RNA-seq data (Supplemental File 3), thereby validating the RNA-seq results. Changes in the expression of four of the nine DEGs were significantly different (p < 0.05) in the qPCR also, as determined by one-way ANOVA analysis (Supplemental File 3).

**Table 2:** Summary of sequencing data obtained for the AgNP/Control-1 and the AgNO3/Control-2 RNA-seq experiments. RNA-seq analyses were performed separately for the two treatments. For each biological repeat, three technical replicates were pooled together prior to library construction. The total number of reads and the percentage of reads mapped per biological replicate are listed.

	Control 1		AgNP		Control 2		AgNO3	
	Number of Reads	Percent Mapped	Number of Reads	Percent Mapped	Number of Reads	Percent Mapped	Number of Reads	Percent Mapped
Repeat 1	33,538,008	94.60%	33,687,566	93.10%	44,432,998	97.28%	53,520,470	97.01%
Repeat 2	27,417,701	89.40%	29,042,251	91.50%	50,463,898	97.25%	47,053,790	97.28%
Repeat 3	26,191,008	87.80%	36,447,804	91.10%	47,893,060	97.60%	53,135,402	99.55%

The FDR-corrected AgNP-induced DEGs included several genes involved in the perception and transduction of stress-related signals, among them PCC1 (AT3G22231), ACD6 (AT4G14400), WAK1 (AT1G21250), ASI-LLP1 (AT5G03350), MIOX4 (AT4G26260), PCR1 (AT1G14880) and three calmodulin-like protein genes (AT2G41090, SAT3G47480, and AT3G50770), all of which were up-regulated in response to AgNP exposure (Fig. 3, Supplemental File 1). Genes involved in biotic stress response also increased in expression in AgNP-exposed plants, among them RING1 (AT5G103800), STMP10 (AT5G44568) and an aspartyl-1 protease (AT5G10760), but several other defense-related genes, including a chitinase (AT243590) and four putatively extracellular heme-peroxidases (AT5G64100, AT1G05250, AT1G49570 and AT5G19890) were transcribed at lower levels (Supplemental File 1). Three genes involved in cell wall organization, namely, two root-specific extensins (AT1G26250 and AT1G26240) and an endoglucanase gene (AT4G16260) also were down-regulated in AgNP-treated plants.

**Fig. 4:**
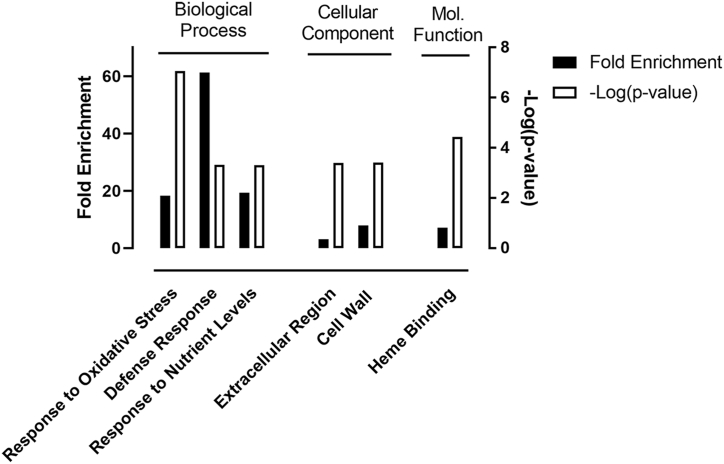
Gene ontology (GO) enrichment analysis of FDR-corrected AgNP-responsive Arabidopsis genes. Filled and open bars represent fold-enrichment and negative Log_10_ of the p-value of enrichment, respectively, in each GO category.

Gene ontology (GO) enrichment analysis (GOrilla [35]) of the FDR-corrected AgNP-altered gene expression pointed to the over-representation of transcripts involved in cell wall metabolism and the signaling and mediation of stress response (Fig. 4 and Supplemental File 4). The highest enrichment of transcripts was found in the stress response category, with gene products involved in cellular defense against ROS and pathogen attack. The cellular components that were highlighted in GO enrichment were the cell wall and the extracellular region. (Fig. 4 and Supplemental File 4). In the set of 426 uncorrected DEGs, two genes encoded for photosynthesis-related genes: LHCB2.4 (AT3G27690), which encodes a light-harvesting chlorophyll *a*/b-binding protein, and ELIP2 (AT4G14690), which encodes a protein involved in regulating the biogenesis of chlorophyll-binding complexes. Both genes were down-regulated more than two-fold. Four additional photosynthesis-related genes, DVR (AT5G18660), PPL1 (AT3G55330), ABC4 (AT1G60600) and STIC2 (AT2G24020), were up-regulated in AgNP-treated plants, but they were not among the DEG set due to their up-regulation being only 1.5- to 1.7-fold.

Of the two FDR-corrected AgNO_3_-induced DEGs, one of them is a gene that encodes the major latex protein-like family protein ZCE1 (AT2G01520). The other gene, AIR1 (AT4G12550), encodes a hypothetical protein induced by auxin and had very low basal expression in control plants (Fig. 3, Supplemental File 2). Neither of these genes were known to be stress-related or belonged to any of AgNP-enriched gene ontology categories. ELIP2 was downregulated in response to AgNO_3_ treatment, but did not pass FDR correction and LHCB2.4 was not among the DEGs.

### 3.4 Chlorophyll concentration

**Fig. 5:**
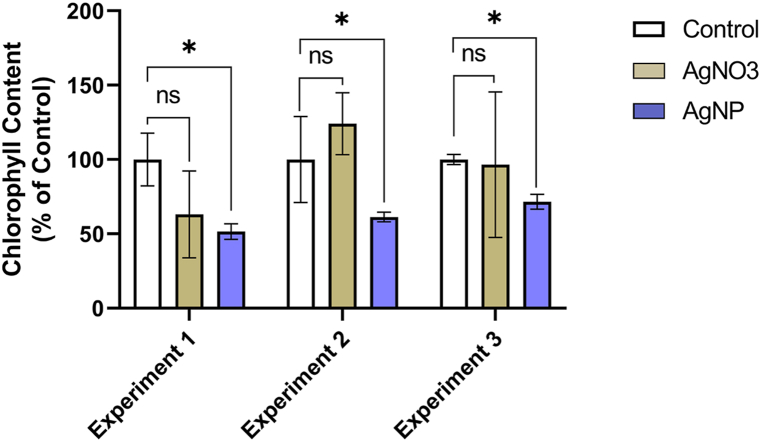
Total chlorophyll concentration in 4 μg/mL AgNP- and 4 μg/mL AgNO_3_-treated Arabidopsis plants as percent of control in three independently repeated experiments. Each column represents the mean of three biological replicates. Error bars: standard error of the mean. ns, non-significant; *, significant at *p* ≤ 0.05 in pairwise comparison.

The AgNP-induced down-regulation of LHCB2.4 suggested that the chlorophyll-content of AgNP-treated plants may be reduced. To test this possibility, we measured chlorophyll concentration on a fresh-mass basis for seedlings grown in AgNP- and AgNO_3_-containing medium, and in control plants. We found that seedlings grown in the AgNP-containing medium had an average chlorophyll concentration of 0.59 mg/g (SE of 0.03) which was 39% lower (p < 0.05) than the 0.97 mg/g mean concentration in control (Fig. 5). Seedlings grown in the AgNO_3_-containing medium, on the other hand, had an average chlorophyll concentration of 0.91 mg/g (SE of 0.13) which was not statistically different from control (Fig. 5). These data pointed to an impact on chlorophyll concentration by AgNP, but not AgNO_3_.

### 3.5 Carbon assimilation rates

The diminishment of chlorophyll concentration in AgNP-exposed plants prompted us to examine photosynthetic efficiency in seedlings grown in AgNP,-, AgNO_3_- and control medium. We registered significant difference (p < 0.05) in various metrics of photosynthetic efficiency on AgNP-treated but not AgNO_3_-treated plants relative to the untreated control plants (Table 3). Carbon assimilation at both growth and saturating PAR (150 μmol m^−2^s^−1^ and 500 μmol/m^2^/s, respectively) was significantly lower in seedlings exposed to AgNPs than in those exposed to AgNO_3_ or in controls (Table 3). Correspondingly, the rate of photosynthetic electron transport was also significantly reduced in AgNP-treated plants. These data demonstrated that photosynthesis is negatively affected by the AgNP, but not by the AgNO_3_ treatment, suggesting the effect is inherently associated with the physical properties of AgNP. Stomatal conductance and transpiration were not significantly affected, eliminating it as a mechanism for the reduced carbon assimilation rates.

**Table 3:** Photosynthetic efficiency in *A. thaliana* seedlings grown in 4 μg/mL AgNP- or 4 μg/mL AgNO_3_-containing medium. Gas exchange rate (Mean ± (SE)) was measured in nine replicates per treatment on 21-day old seedlings. Each replicate was tested between 3 and 6 h after the start of the diurnal light period. Means that are not followed by the same letters, within a row, are significantly different (*P* < 0.05).

Gas Exchange Metrics	Response to AgNP		Response to Ag^+^	
	Control	AgNP	Control	AgNO3
aA_amb_ (μmol CO_2_ m^−2^ s^−1^)	4.54 ± 0.32^a^	1.96 ± 0.13^b^	4.66 ± 0.17^c^	4.50 ± 0.25^c^
bA_sat_ (μmol CO_2_ m^−2^ s^−1^)	5.92 ± 0.46^a^	2.59 ± 0.17^b^	5.99 ± 0.25^c^	5.97 ± 0.28^c^
cJmax (μmol m^−2^ s^−1^)	43 ± 4.38^a^	21 ± 2.25^b^	51 ± 5.08^c^	48 ± 5.25^c^
dΦ	0.43 ± 0.04^a^	0.33 ± 0.05^a^	0.52 ± 0.07^c^	0.49 ± 0.08^c^
eVcmax (μmol m^−2^ s^−1^)	56 ± 1.80^a^	45 ± 2.62^b^	47 ± 2.22^c^	45 ± 2.62^c^
fJ (μmol m^−2^ s^−1^)	64 ± 1.96^a^	39 ± 2.12^b^	66 ± 2.31^c^	56 ± 2.52^c^
gTPU (μmol m^−2^ s^−1^)	4.4 ± 0.21^a^	2.8 ± 0.16^b^	5.5 ± 0.15^c^	5.4 ± 0.21^c^

a A_amb_: Carbon assimilation rate at growth PAR (150 μmol/m^2^/s).

b A_sat_: Carbon assimilation rate at saturating PAR (500 μmol/m^2^/s).

c J_max_: Maximum rate of electron transport at saturating light.

d Φ: Initial slope of J.

e V_cmax_: maximum carboxylation rate.

f J: photosynthetic electron transport at the measured light intensity.

g TPU: triose phosphate use.

## 4 Discussion

### 4.1 AgNPs and ionic silver trigger different responses in *Arabidopsis thaliana*

Under aqueous conditions, AgNPs release silver ions [36], which are phytotoxic at high concentrations, [11,12]. Knowing whether the cause of toxicity is inherent to the physical formulation of AgNPs or caused by silver ions leached from their surface, or both, is key to understanding the molecular mechanism by which nanosilver affects plants. To address this question, we exposed Arabidopsis seedlings to low levels of AgNPs or AgNO_3_. To limit Ag ^+^ leaching from the nanoparticles, we maintained the pH of the growth medium at 7.0 by the incorporation of MOPS buffer, as lower pH increases Ag + leaching [23]. Under these conditions, comparable amounts of Ag accumulated in plants of both treatments (Fig. 2, Table 1). Despite the similar levels of Ag concentrations in the AgNP- and AgNO_3_-exposed plants, we detected considerably different patterns of transcriptome changes between the two treatments (Fig. 3). In AgNP-treated plants, changes occurred in transcripts involved in stress-response and cell wall modification, as well as the light harvesting chlorophyll *a*/b-binding protein LHCB2.4, which encodes a key component of the photosynthetic apparatus. The expression of LHCB2.4, was reduced by nearly 2.5-fold in AgNP-exposed plants. In response to AgNO3-exposed plants, the expression of these genes remained unchanged.

The AgNP-induced reduction in LHCB2.4 expression prompted us to measure chlorophyll concentration and carbon assimilation rates. We found that chlorophyll concentration was reduced by 39% (p < 0.001) and carbon assimilation rates were reduced by as much as 56.9% (p < 0.05) in AgNP-treated seedlings. We found that in AgNO_3_-exposed plants, on the other hand, these indices remained statistically indistinguishable from those in the control plants (Fig. 5 and Table 3). The reduction in carbon assimilation rates along with the drop in both LHCB2.4 expression and chlorophyll content indicated that AgNPs impeded photosynthesis in Arabidopsis. Stomatal conductance was not a limiting parameter to carbon assimilation, indicating that the other limiting parameters in Table 3 are directly affected by AgNPs. The impact of AgNPs on chlorophyll content is an overarching theme in the silver nanotoxicty literature. Nair and Chung [37] and Qian et al. [26] measured approximately 50% and 37% reduction of total chlorophyll concentration in Arabidopsis grown in medium containing AgNP at 1 μg/mL and 3 μg/mL, respectively. Song et al. [38] and Nair and Chung [9] measured similar levels of reduction in chlorophyll content in nanosilver-treated tomato and rice, respectively. Decline in chlorophyll content by AgNPs was also reported in the alga *Chlamydomonas acidophila* [34] and the aquatic plant *Ottelia alismoides* [39]. Transmission electron microscopic (TEM) observations in various plant species suggest that one of the mechanisms underpinning the attenuation of photosynthesis is structural damage to the chloroplast. Using TEM, Qian et al. [26] and Stefanic et al. [40] have observed altered thylakoid membranes in AgNP-exposed Arabidopsis and tobacco plants, respectively. Wang et al. [39] also reported AgNP accumulation in and damage to chloroplast ultrastructure in *O. alismoides*. In agreement with our findings, others also observed a reduction in photosynthetic efficiency. Sosan et al. [41] measured 30%–58% reduction in Fv/Fm values in Arabidopsis when the plants were grown in the presence of high concentrations (500–3000 μg/mL) of AgNPs, indicating that the quantum efficiency of photosystem II was dramatically reduced. Jiang et al. [13] found that AgNPs inhibited the thylakoid electron transport chain which reduced both photosynthetic efficiency and the ability of the plant to protect itself from high light. It is possible therefore that the deterioration of photosynthetic efficiency in AgNP-treated plants is caused by damage to the chloroplast and/or decline in chlorophyll content. Our data lends further support to this interpretation. AgNPs reduced J_max_ (Table 3) and therefore likely inhibited NADPH production, which would result in inefficient carbon fixation within the Calvin cycle. This inefficiency is reflected by the A/Ci analysis, indicating that Rubisco regeneration, and TPU activity are all inhibited in AgNP-exposed plants [28].

### 4.2 The AgNP-affected transcriptome implies activation of defense to offset stress

To gain insight into the transcriptomic changes underlying AgNP impact on Arabidopsis, we performed an RNA-seq analysis to identify transcripts that changed in abundance in response to AgNP exposure. To reduce the probability of type-I error, we used true biological repeats which enabled us to glean those transcripts that most consistently changed in response to AgNP exposure. We detected 426 and 278 genes that significantly altered their expression in response to AgNP- and AgNO_3_-treatment, respectively. These two DEG sets overlapped only by 39 genes, (Fig. 3). Previous microarray-based transcriptomic data corroborate our results: Kaveh et al. [10] identified 375 and 141 Arabidopsis DEGs in response to AgNPs and AgNO_3_, with 44 genes responding to both, indicating considerable difference between the impact caused by particulate metallic and Ag ^+^ ionic forms of silver on the transcriptome in Arabidopsis. Proteomic analysis in tobacco found a much greater concordance between the AgNP and the AgNO_3_ response: between sets of 58 AgNP- and the 67 AgNO_3_-responsive proteins, as many as 55 overlapped [27].

To further reduce the possibility of type-I error, we focused our analysis on DEGs that passed FDR correction at p ≤ 0.05. GO enrichment analysis of the FDR-corrected gene set demonstrated that AgNPs affected cell wall metabolism and triggered a stress response that had salicylic acid-mediated pathogen defense-like features (Fig. 4). Despite the defense-like gene expression pattern, we find it unlikely that AgNP-induced stress may be perceived by the plant as an attack by an obligate pathogen, because transcript levels of such key defense response regulators as NPR1, EDS1, PAD4, SAG101 and LSD1 remained unchanged in AgNP-treated plants (Supplemental File 1). It is more likely that the immune-like response may have been the corollary of stress-signaling pathways. A potential link between the signs of altered cell wall biogenesis and activated pathogen defense is suggested by the approximately 4-fold up-regulation of the cell wall-associated oligogalacturonide-binding kinase WAK1 (AT1G21250) (Supplemental File 1), which had been shown to play a key role in initiating pathogen attack signals at the cell wall [42].

GO enrichment analysis of DEGs also demonstrated that AgNP-treated Arabidopsis plants responded to changes in redox balance (Fig. 4). The expression of the ROS-responsive calmodulin-like gene-10 (CML10, AT2G41090), a key positive regulator of ascorbate biosynthesis via the Smirnoff–Wheeler pathway, increased about 3-fold, and the expression of the myo-inositol oxygenase gene (MIOX4, AT4G26260), encoding the first step in ascorbate synthesis from myo-inositol, increased 6-fold. This suggested a significant up-regulation of ascorbate synthesis, a key scavenger of hydrogen peroxide in the apoplast and the chloroplast. This is in agreement with commonly reported observations that AgNPs cause a shift in the redox balance toward oxidative conditions, as documented by experimental measurements of an increase in reactive oxygen species (ROS) [9,12,13,37,41,43,44]. In qPCR-based gene expression measurements, Qian et al. [26] and Nair and Chung [9] documented the up-regulation of catalase and several members of the superoxide dismutase and ascorbate peroxidase gene families in Arabidopsis and rice, respectively. Microarray results by Kaveh et al. [10] have documented the elevated expression of a peroxidase and two superoxide dismutase genes in AgNP-exposed Arabidopsis.

### 4.3 Gene expression patterns suggest AgNP-triggered interference with cell wall metabolism

Extracellular heme-peroxidases featured prominently in GO enrichment analysis. Their regulation was responsible for the overrepresentation of the extracellular region in the cellular component GO category (Supplemental file 4). Previously, experimental evidence has been provided that two of them, namely PRX64 (AT5G64100) and PRX2 (AT1G05250), are cell wall-bound [45] and that the latter plays a role in altering cell wall structure [46]. In addition to these peroxidases, we identified, in the FDR-corrected DEG set, two root-specific proline-rich extensin transcripts whose protein products act on cell wall constituents. These transcripts, namely AT1G26240 and AT1G26250, decreased 18- and 6-fold in the AgNP-treated plants, respectively (Supplemental File 1). In addition, in the non-FDR-corrected DEG set, six additional proline-rich extensin genes and a leucine-rich extensin gene were down-regulated approximately 3-fold (Supplemental File 1). Transcripts for cell wall polysaccharide-modifying enzymes such as a beta-1,3-glucanase (AT4G16260) and two pectin methylesterases (AT1G14890 and AT5G38610) were down-regulated, as well as the gene encoding polygalacturonase-inhibiting protein 1 (AT5G06860) (Supplemental File 1). It is reasonable to speculate, therefore, that the down-regulation of these genes indicate an interference with cell wall metabolism by AgNPs, which were previously shown to accumulate in the apoplast along the cell wall and cause damage in Arabidopsis [47,48]. AgNP-inflicted cell wall damage, specifically at the root, has been studied previously. Mirzajani et al. [49] showed that in rice, AgNPs penetrated the cell wall, altering cell morphology. Speranza et al. [12] observed deformed pollen grains and abnormal membrane structures beneath the wall of AgNP-treated Kiwifruit pollen. The impacts were attributed to elevated apoplastic hydrogen peroxide levels, nanoparticle aggregation in the cell wall and interference with cytoplasmic transport proteins [12,47,48].

### 4.4 Down-regulation of peroxidase genes is unique to our experimental system

While our GO enrichment results were suggestive of a disturbance in redox balance, they were not equivocally in agreement with observations from previous studies. A notable disagreement between our results and those of others was in the expression of peroxidase genes. While Nair and Chung, Kaveh et al. [10], and Qian et al. [26] reported AgNP-induced increase in peroxidase expression, we observed the down-regulation of four heme-peroxidase genes. In addition, we registered a 4.5-fold decrease in the expression level of transcription factor UBP1 (AT2G47270), which controls the balance of ROS in roots by regulating the expression of peroxidase genes [50], (Supplemental File 1). The reason for this discrepancy may be our highly stringent experimental design. While our reliance on true biological repeats mitigated type-I error, it also increased the probability that we failed to identify genes that changed in response to treatment in a less reproducible manner (type-II error).

Another reason for this discrepancy may be due to the unique features of our experiment, most notably, the use of a biological buffer to stabilize pH at 7.0 in the growth medium. We observed that the 20 nm diameter silver nanoparticles formed aggregates of 82.6 ± 43.2 nm when mixed with our culture medium (Fig. 1). Such aggregation has also been shown by Oukarroum et al. [34] who suspended AgNPs of 50 nm diameter in algal growth medium and observed that AgNPs formed aggregates of 132.8 and 215.5 nm in medium pH-adjusted at pH 4.0 and pH7.0, respectively. They observed that AgNPs had a considerably lower toxicity at pH 7.0 and caused a less significant reduction in chlorophyll content than at pH 4.0. These data indicated that the phytotoxicity of AgNPs is influenced by medium pH, likely through the modified physicochemical properties of the particles. This is supported by recent data from Biba et al. [44] who used AgNPs with polyvinylpyrrolidone (PVP) and cetyltrimethylammonium bromide (CTAB) coating and found a greater impact of the latter on the rise of ROS, the activation of antioxidant response and the abundance of proteins involved in stress-response and energy metabolism in tobacco. Stefanic et al. [27] studied AgNPs coated with CTAB, PVP, and citrate, and found that citrate-coated AgNPs had the least impact on the chlorophyll and carotenoid content of and photosynthetic efficiency in tobacco. Clearly, the physical properties of the AgNPs (shape, size, coating and aggregation) and the conditions under which they are applied (pH, ionic strength and the buffering capacity of the growth medium), and the species and age of target plants have a major influence on the impact of nanosilver. This ultimately makes comparison across various experiments difficult.

Our results are in agreement with data generated from broad range of plants including Arabidopsis [10], tobacco [44], mustard [11], arugula [51], kiwifruit [12], trumpetweed [52], pokeweed [52] and *O. alismoides* [39]. Notable exceptions are to this are the duckweed species *Lemna minor* [25] and *Spirodela polyrhiza* [13] in which AgNO3 was reported to cause a more severe toxic impact than AgNPs. Despite the differential response in duckweed species, there is an emerging paradigm that the impact of AgNPs cannot be solely attributed to the presence of silver ions, but is inherently linked to the particulate nature of nanosilver.

Despite the large body of literature, the exact molecular mechanism through which particulate silver causes phytotoxicity still remains to be unveiled. Taken together all the data presented by us and others, two models of possible mechanisms emerge. The first potential scenario is physical damage to the cell wall, and the cytoplasmic and organelle membranes of the cell. According to this possibility, AgNPs, taken up as particles by the plant [8], disrupt the integrity of subcellular structures [[12], [13], [14]], which then leads to physiological dysfunction. The second potential scenario is that the dissociation of Ag^+^ ions from nanoparticles inside the plant creates elevated Ag^+^ concentrations which then interfere with catalytic reactions and the functioning of macromolecules. According to this possibility, nanoparticles serve as highly effective delivery vehicles of metallic silver into the apoplast and symplast, and the ultimate causal agents of toxicity are still the Ag^+^ ions dissociated from them. A potential reason why this level of toxicity cannot be recreated with direct exposure of the plants to silver ions is that Ag^+^ is readily oxidized to Ag_2_O in the growth medium. Because the rate of dissolution of Ag_2_O to Ag^+^ is lower than that of Ag^0^, internalized Ag_2_O cannot create the same level localized Ag ^+^ concentrations as AgNPs. It is important to note that these two possibilities are not mutually exclusive, as internalized AgNPs can cause damage by both structural disruption and localized Ag^+^ release simultaneously.

The hypothesis that AgNP toxicity is associated with its lower degree of oxidation is testable and a worthwhile direction for future investigations. Comparative analysis of silver toxicity mechanisms in Arabidopsis and a duckweed species could also help unveil the mechanism of AgNP toxicity. Further research in this area is needed if agrochemical application of nanosilver is to be safely implemented. While a series of recent papers advocate for the use of AgNPs at low concentrations as stimulants on crops [3,[53], [54], [55]], field applications at even very low levels may harm non-target organisms. Therefore, a thorough understanding of the molecular mechanism underlying silver phytotoxicity is much needed.

## Ethics approval

Review and/or approval by an ethics committee was not needed for this study because we conducted our testing on the plant *Arabidopsis thaliana*. No animal or human subjects were involved in the study.

## Data availability statement

All RNA-seq data has been made publicly available through the GEO repository of the National Center for Biotechnology Information (https://www.ncbi.nlm.nih.gov/geo/, AgNP accession: GSE163583 and AgNO3 accession: GSE198360). Additional data will be made available upon request.

## CRediT authorship contribution statement

**Vincent Mays:** Writing – review & editing, Writing – original draft, Methodology, Investigation, Conceptualization. **Natalie Smith:** Writing – review & editing, Writing – original draft, Visualization, Validation, Investigation, Data curation. **Cody Pham:** Writing – review & editing, Writing – original draft, Visualization, Validation, Investigation, Data curation, Conceptualization. **Margaret White:** Writing – review & editing, Writing – original draft, Visualization, Validation, Investigation. **Qihua Wu:** Writing – review & editing, Methodology, Investigation. **Alexander Linan:** Writing – review & editing, Writing – original draft, Methodology, Conceptualization. **Jacob Berry:** Writing – review & editing, Methodology, Investigation, Data curation. **D. Alexander Wait:** Writing – review & editing, Writing – original draft, Methodology, Formal analysis, Data curation, Conceptualization. **Laszlo Kovacs:** Writing – review & editing, Writing – original draft, Supervision, Project administration, Funding acquisition, Data curation.

## Declaration of competing interest

The authors declare that they have no known competing financial interests or personal relationships that could have appeared to influence the work reported in this paper.
